# Draft Genome Sequence of *Chromatium okenii* Isolated from the Stratified Alpine Lake Cadagno

**DOI:** 10.1038/s41598-018-38202-1

**Published:** 2019-02-13

**Authors:** Samuel M. Luedin, Nicole Liechti, Raymond P. Cox, Francesco Danza, Niels-Ulrik Frigaard, Nicole R. Posth, Joël F. Pothier, Samuele Roman, Nicola Storelli, Matthias Wittwer, Mauro Tonolla

**Affiliations:** 1 Microbiology Unit, Department of Botany and Plant Biology, University of Geneva, Geneva, Switzerland; 2 Laboratory of Applied Microbiology, Department of Environment, Constructions and Design, University of Applied Sciences of Southern Switzerland (SUPSI), Bellinzona, Switzerland; 3 Biology Division, Spiez Laboratory, Federal Office for Civil Protection, Spiez, Switzerland; 4 Interfaculty Bioinformatics Unit, University of Bern, Bern, Switzerland; 5 Graduate School for Cellular and Biomedical Sciences, University of Bern, Bern, Switzerland; 6 Department of Biochemistry and Molecular Biology, University of Southern Denmark, Odense, Denmark; 7 Department of Biology, University of Copenhagen, Helsingør, Denmark; 8 Department of Biology, University of Southern Denmark, Odense, Denmark; 9 Department of Geosciences and Natural Resource Management (IGN), University of Copenhagen, Copenhagen, Denmark; 10 Environmental Genomics and System Biology Research Group, Zurich University of Applied Sciences (ZHAW), Wädenswil, Switzerland; 11 Alpine Biology Center Foundation, Bellinzona, Switzerland

## Abstract

Blooms of purple sulfur bacteria (PSB) are important drivers of the global sulfur cycling oxidizing reduced sulfur in intertidal flats and stagnant water bodies. Since the discovery of PSB *Chromatium okenii* in 1838, it has been found that this species is characteristic of for stratified, sulfidic environments worldwide and its autotrophic metabolism has been studied in depth since. We describe here the first high-quality draft genome of a large-celled, phototrophic, *γ*-proteobacteria of the genus *Chromatium* isolated from the stratified alpine Lake Cadagno, *C*. *okenii* strain LaCa. Long read technology was used to assemble the 3.78 Mb genome that encodes 3,016 protein-coding genes and 67 RNA genes. Our findings are discussed from an ecological perspective related to Lake Cadagno. Moreover, findings of previous studies on the phototrophic and the proposed chemoautotrophic metabolism of *C*. *okenii* were confirmed on a genomic level. We additionally compared the *C*. *okenii* genome with other genomes of sequenced, phototrophic sulfur bacteria from the same environment. We found that biological functions involved in chemotaxis, movement and S-layer-proteins were enriched in strain LaCa. We describe these features as possible adaptions of strain LaCa to rapidly changing environmental conditions within the chemocline and the protection against phage infection during blooms. The high quality draft genome of *C*. *okenii* strain LaCa thereby provides a basis for future functional research on bioconvection and phage infection dynamics of blooming PSB.

## Introduction

**Figure 1:**
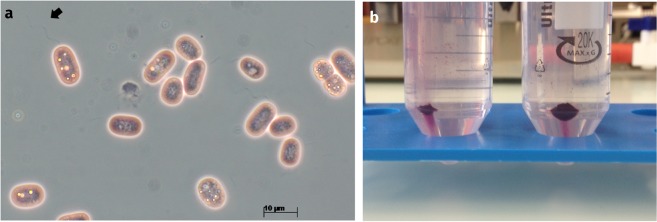
Visual characterization of *Chromatium okenii* strain LaCa. (**a**) Microscopic image of *C*. *okenii* str. LaCa cells enriched form water sample from Lake Cadagno take at 12 m depth. Intracellular sulphur globules are visible as yellow, highly-refractive spheres. The polar flagellar tuft is visible (black arrow). Cells were directly mounted on a microscopy slide in 0.9% NaCl solution. An Axio Imager 2 microscope (Carl Zeiss Microscopy GmbH, Germany) with a EC Plan NEOFLUAR objective (100x, phase contrast) and an AxioCam MRc Rev3 digital camera were used to take photomicrographs. Images were processed with the AxioVision SE64 v4.8.2 software suite. (**b**) Visible *C*. *okenii* cell pellets enriched from a water sample from Lake Cadagno after 10 min centrifugation at 15 *g*.

*Chromatium okenii*, belonging to the purple sulfur bacteria (PSB, family Chromatiaceae), was described in the environment as massive purple blooms as early as 1838 by the microbiologists Ehrenberg and Weisse as: “*Monas corpore cylindrico*, *aequabili*, *parumpcr curvato*, *ter quaterve longiore quam lato*, *utrinque rotundato*, *1/192 lineae attingens*, *volutando procedens*, *vacillans*, *rubra; socialis*”^1^, and was subsequently characterized in more detail by Maximilian Perty in 1852^2^ and Sergei Winogradsky in 1888^3^. Light microscopy was used to study changes in microbial motility patterns under opposing gradients of light and sulfur^4,5^. *C*. *okenii* thereby showed scotophobotaxis – i.e. the sudden reorientation of a moving microorganism when experiencing a decrease in light intensity over time – negative aerotaxis and positive chemotaxis towards H_2_S *in vitro*^6–8^. Populations of *C*. *okenii* have since been found in freshwater ecosystems worldwide such as lakes, ponds and bacterial mats in bogs^9–13^. Schlegel and Pfennig managed to isolate a *C*. *okenii* strain later designated DSM 169^T^ from a pond in Germany in 1960^14^, however the type strain got lost over time. *C*. *okenii* is described as rod-shaped, 5.0 to 6.5 *µ*m wide and 8.0 to 10.0 *µ*m long, stains Gram-negative, contains okenone and bacteriochlorophyll *a* (BChl *a*) as the main photosynthesis pigments, and is motile through flagella (Fig. 1a)^15^. The cells contain intracellular carbon storage compounds, such as polyhydroxybutyrate (PHB), glucose, glycogen, polyphosphate and also elemental sulfur globules that typically functions as an electron donors for anaerobic photosynthesis in PSB^16^.

The meromictic Lake Cadagno in the Swiss Alps (46°32′59″N 8°42′41″E) is a prime example of a stratified, sulfidic environment where anoxygenic phototrophic sulfur bacteria of the families Chromatiaceae (purple sulfur bacteria; PSB), such as *Chromatium*, *Thiodictyon*, *Lamprocystis* and *Thiocystis* spp. and Chlorobiaceae (green sulfur bacteria; GSB) of the *Chlorobium* genus, thrive to dense populations during the summer months. At a depth of around 12 m, sulfide of a concentration of approximately 0.2 mM and the relative light availability of around 5 *µ*mol quanta m^−2^ s^−1^ allows this heterogeneous community to grow up to 10^7^ cells mL^−1^ by anoxygenic photosynthesis^17,18^. The presence of an at least 9,450 year old *Chromatium* sp. 16S rRNA-gene sequence in Lake Cadagno sediments has been demonstrated^19^ and in recent years a unique *C*. *okenii* strain has been detected using a combination of fluorescent *in situ* hybridization (FISH) and 16S rRNA gene analysis^20–22^. Whereas *C*. *okenii* represents only 1–10% of all bacterial cells and 22–83% of the phototrophic community^21–25^, due to its large size it comprises to 72% of the total biovolume of the microbial chemocline population^24^. Large seasonal variations of *C*. *okenii* were observed with concentrations ranging from 10^4^–10^6^ cells mL^−1^. When cell concentrations were monitored using FISH and flow cytometry in Lake Cadagno, *C*. *okenii* dominated over small celled PSB from July to September that was then followed by a *C*. *okenii* population decline within two weeks in October^21,24^. However, due to a mixing event in 2000 and the following massive bloom of the *Chlorobium clathratiforme*, increasing the total number of phototrophic sulfur bacteria three-fold between 2000–2005, also less regular *C*. *okenii* population dynamics were observed^26^, suggesting that environmental influences may have a long lasting impact on microbial community composition^26^. With a doubling time of 5 to 7 days, *C*. *okenii* was found to assimilate up to 70% of total carbon and 40% of ammonium with light^27,28^. Additional evidence was given by analyses of bulk carbon isotope fractionation (between δ^13^C_POC_ and δ^13^C_DIC_) in the Cadagno chemocline, in which 36% and 52% of the total bulk δ^13^C -signal was attributed to *C*. *okenii* in October and June, respectively^29^. As grazing on *C*. *okenii* by the ciliate *Trimyema compressum* was shown *in vitro*^30^, *C*. *okenii* may also function as a major food source for zooplankton in Lake Cadagno.

The importance of *C*. *okenii* in bioconvection in Lake Cadagno has been discussed theoretically^31^. Interestingly, a spatial and temporal correlation of convection in zones with high concentrations of *C*. *okenii* (10^5^–10^6^ cells mL^−1^) was lately inferred *in situ*^32^. Short-time dynamics in sulfide uptake of *C*. *okenii* and putative interactions between *C*. *okenii* and the GSB *Chlorobium phaeobacteroides* were also demonstrated recently^22^. And in another novel *in situ* study by Berg *et al*. on *C*. *okenii* in Lake Cadagno, it was found that anaerobic sulfide oxidation is coupled to aerobic respiration using sulfide as electron acceptor by active vertical movement from the oxic to anoxic parts of the water column and back^33^.

Herein, we provide the first annotated high-quality draft genome for a member of a large celled PSB genus *Chromatium*, namely *C*. *okenii* strain LaCa, enriched from Lake Cadagno. Importantly, the available sequence data on PSB and GSB isolates of Lake Cadagno enabled us to first, compare core-genomes and, secondly to further elucidate on strain-specific biological functions. The successful enrichment and the high quality draft genome of *C*. *okenii* strain LaCa are fundamental for a detailed understanding of nutrient fluxes and microbial interactions within the balanced ecosystem of the Lake Cadagno chemocline and is an important addition to our understanding of global microbial sulfur cycling.

## Material and Methods

The complete materials and methods section has previously been described in Luedin 2018^34^.

### Chemicals

All chemicals were purchased from Sigma-Aldrich AG (Buchs, Switzerland), if not further specified.

### Enrichment

Physicochemical measurements on Lake Cadagno were made with an YSI 6000 profiler (Yellow Springs Inc., Yellow Springs OH, USA) on 14 July 2016. In order to understand carbon isotope fractionation of PSB and GSB strains, *C*. *okenii* was previously enriched to high purity by sedimentation and dilution, however cultivation was not established^29^. We used a comparable approach for this study. Samples were collected with a 1 L *Ruttner*-type sampling bottle (Hydrobios Apparatebau GmbH, Germany) taken at depths with maximum turbidity and rapid changes in redox-potential between 11.6–12.0 m, indicating a dense bacterial population at the chemocline, as previously described^31^. The bottles were brought immediately to the laboratory and were placed at natural illumination (2000 lux PAR, or about 36 *µ*mol quanta m^−2^ s^−1^) at 16 °C for 6 hours. The purple precipitates thus obtained (Fig. 1b) were identified by cell morphology with light microscopy as *Chromatium* sp. based on previous descriptions in the literature (e.g. ref.^35^). Other bacteria were also present, however only in low numbers (<10%). Cells were collected with a 10 mL pipette and transferred to 50 ml tubes. The cells were then centrifuged 10 min at 15 *g* at room temperature (RT). The supernatant was carefully discarded, and the residual 10 mL were collected and combined in 100 ml serum bottles. The bottles were then filled up with filtered (0.45 *µ*m) chemocline water. A rubber plug was applied, and a vacuum was generated with a suction pump to remove O_2_. Using a syringe, 100 *µ*L of a 35 mM Na_2_S 9 H_2_O solution was added to each sample, resulting in a final concentration of 0.03 mM. Subsamples were conserved at −20 °C before DNA extraction.

### Light Microscopy

Cells were directly added directly to on the microscopy slides in 0.9% NaCl solution. An Axio Imager A.2 microscope (Carl Zeiss Microscopy GmbH, Germany) with an EC Plan NEOFLUAR objective (100×, phase contrast) was used to examine the enrichments. Images were taken with an AxioCam MRc Rev3 digital camera and images were processed with the AxioVision SE64 v4.8.2 software suite (Carl Zeiss Microscopy GmbH).

### DNA Extraction, Sequencing and Genomic Analysis

Frozen samples were thawed on ice and cells were collected by centrifugation for 15 min at 10,600 *g*. Genomic DNA (gDNA) was extracted with phenol/chloroform/isoamylalcohol solution (25:24:1, *v*/*v*) adhering to the protocol provided by *Pacific Biosciences* in combination with phase lock gels for phase separation (VWR International, Radnor, USA). gDNA was concentrated and washed using *AMPure* beads (Agencourt, Beckman Coulter Life Sciences, Indianapolis, USA) following the E2612 protocol from New England Biolabs^36^. Concentration of the DNA was assessed using a *Qubit* UV/VIS absorption reader (Thermo Fisher Scientific, Rheinach, Switzerland).

The library construction and Single-Molecule Real-Time sequencing (SMRT) was done on the *Pacific Biosciences RS II* platform at the Functional Genomic Center Zurich, Zurich, Switzerland. A 10 kb *SMRTbell* library was constructed using the DNA Template Prep Kit 1.0 (Pacific Biosciences, Menlo Park, USA). *SMRTbell* template fragments over 10 kb length were used for creating a *SMRTbell*-Polymerase Complex with P6-C4 chemistry (Pacific Biosciences) according to the manufacturer instructions. Two *SMRT* cells v3.0 (Pacific Biosciences) for *PacBio RS II* chemistry were used for sequencing. Sequencing quality reports were created through the *SMRT* portal software.

*PacBio RSII* reads were assembled using the *canu* assembler v1.4, [RRID:SCR_015880]^37^. Genes were annotated using the NCBI Prokaryotic Genome Annotation Pipeline *GeneMarkS*+, v4.3 [RRID:SCR_011930]. *PhiSpy* v2.3^38^, PHASTER^39^ and *VIRSorter* v1.0.3^40^ were used to detect phage and prophage related sequences. *EggNOG* [RRID:SCR_002456]^41^ was used to classify the predicted genes into COG-categories and *OrthoVenn*^42^ was used to classify gene families and visualize clustering. Genes were to assigned to *KEGG* categories with the *blastKOALA* v2.1 [RRID:SCR_012773]^43^. *AmphoraNet* [RRID:SCR_005009]^44^, *BUSCO* [RRID:SCR_015008]^45^ and *CheckM* v1.0.12 [RRID:SCR_016646]^46^ were used to asses genome completeness and contamination. *Centrifuge* v1.0.3^47^ was used to classify the PacBio raw reads against the NCBI nt database (including additional genomic sequences of *Lamprocystis* strain CadA31 and “*T*. *syntrophicum”* strain Cad 16^T^). The CRISPR-CAS++ v1.0.5^48^ web server was used to infer Clustered regularly interspaced short palindromic repeats (CRISPR) and associated proteins (Cas) in the *C*. *okenii* genome. CRISPR-Cas arrays with an evidence level of 1 were excluded from the analysis.

### Phylogenetic Analysis

*Roary*^49^ was used to compare the core genomes of sequenced *Chromatiaceae*. Out of this dataset, 100 single-copy orthologues were selected randomly and their sequences aligned with *MUSCLE*^50^. The best-fit phylogenetic model and subsequent consensus tree estimation, based on maximum-likelihood and 1,000 bootstrap iterations, was performed with the *W*-*IQ-TREE* v1.6.3 platform^51^.

A *C*. *okenii* strain LaCa full length 16S rRNA gene sequence (CXB77_RS15475) was used to search 16S rRNA gene NCBI database for related sequences with *BLASTn* [RRID:SCR_001598]. The online tool *W*-*IQ-TREE* v1.6.3^52^ was used to create the phylogenetic trees based on the alignment with *MAFFT* v7 215^53^ [RRID:SCR_011811]. A combination of 1,000 bootstrap iterations and 1,000 aLRT replications were performed. *FigTree v1*.*4*.*3*^54^ was used to render phylogenetic trees.

## Results and Discussion

### Genomic Features and Phylogeny

The *de novo* sequencing of an enrichment of *C*. *okenii* strain LaCa was successfully done with a PacBio RSII system using two SMRTcells. A total of 45 contigs were assembled with a total length of 3,784,749 bp, a N_50_ of 448,938 bp and a L_50_ of 3. The GC content was found to be 49.8% (Table 1). More details on the sequencing output can be found under Supplementary Figure S1a and a classification of *C*. *okenii* strain LaCa is given by Supplementary Table S1. The chromosome comprises three long contigs (PPGH01000034.1, PPGH01000035.1 and PPGH01000037.1) with coverage values between 24× and 27× (Supplementary Fig. S1b). Further five contigs (PPGH01000013.1, PPGH01000018.1, PPGH01000010.1, PPGH01000038.1 and PPGH01000036.1) were found to be associated by partial sequence overlaps (Supplementary Fig S1b) and showed an average coverage of 25× (22–27×). Due to repetitive sequences of >20 kb we could not circularize the chromosome and therefore created a pseudo-circular map with contig borders indicated (Supplementary Fig. S1c). Additional four putative circular sequences (PPGH01000033.1, PPGH01000043.1, PPGH01000024.1 and PPGH01000027.1) were identified (Supplementary Fig. S1b). The other 33 shorter contigs with coverage <22× showed partial or complete overlap with the seven longest contigs.

**Table 1:** Genome statistics for *Chromatium okenii* strain LaCa.

Attribute	Value	% age of total
Genome size (bp)	3,784,749	100.0
DNA coding (bp)	2,686,967	71.0
DNA G + C (bp)	1,884,805	49.8
DNA scaffolds	45	100.0
Total genes	3,792	100.0
Protein coding genes	3,016	79.5
RNA genes	67	1.8
rRNA genes	3, 3, 3 (5S, 16S, 23S)	0.2
tRNA genes	53	1.4
ncRNA genes	5	0.1
Pseudo genes	708	18.7
Genes in internal clusters	NA	NA
Genes with function prediction	1,726	45.5
Genes assigned to COGs	2,291	60.4
Genes KEGG predictions	1,335	35.2
Genes with Pfam domains	2,274	60.0
Genes with signal peptides	163	4.3
Genes with transmembrane helices	556	14.7
CRISPR repeats	2	0.1

The genome of *C*. *okenii* strain LaCa was considered as a high quality draft due to the high number of single copy genes when analysed with *BUSCO*; 129 complete/single copy genes, *amphoraNet*; 40 genes homologous to *Allochromatium vinosum* and CheckM; 88.89% (490 of 545) marker genes specific for Chromatiaceae, respectively. The initial assembly was then reduced to the seven longest contigs and the linking contig PPGH01000036.1 and repeated completeness and contamination analysis was repeated using CheckM. Completeness was thereby found to be 88.86%, whereas contamination could be reduced to 1.11%. Additionally, the number of multiple marker genes was reduced from 56 to 8 (Supplementary Tables S2 and S3 and Supplementary Fig. S2), and tetranucleotide frequency and GC content were found to be uniform amongst the seven longest contigs (Supplementary Fig. S3). Mapping of raw PacBio reads to the NCBI nt database and other sequenced PSB isolates from Lake Cadagno revealed that 96% of all reads assigned to Chromatiales mapped on the *C*. *okenii* strain LaCa contigs (Supplementary Table S4).

COG classification of the complete dataset of the 3,016 protein coding genes resulted in 2,022 assigned proteins (Table 2).

**Table 2:** Clusters of Orthologous Genes (COG) functional categories of *Chromatium okenii* strain LaCa.

Code	Value	Percentage %	Description
J	132	4.38	Translation, ribosomal structure and biogenesis
A	2	0.07	RNA processing and modification
K	67	2.22	Transcription
L	182	6.03	Replication, recombination and repair
B	1	0.03	Chromatin structure and dynamics
D	39	1.29	Cell cycle control, Cell division, chromosome partitioning
Y	0	0.00	Nuclear structure
V	61	2.02	Defence mechanisms
T	232	7.69	Signal transduction mechanisms
M	117	3.88	Cell wall/membrane biogenesis
N	51	1.69	Cell motility
Z	0	0.00	Cytoskeleton
W	0	0.00	Extracellular Structures
U	60	1.99	Intracellular trafficking and secretion
O	82	2.72	Posttranslational modification, protein turnover, chaperones
X	0	0.00	Energy production and conversion
C	120	3.98	Energy production and conversion
G	55	1.82	Carbohydrate transport and metabolism
E	75	2.49	Amino acid transport and metabolism
F	39	1.29	Nucleotide transport and metabolism
H	74	2.45	Coenzyme transport and metabolism
I	31	1.03	Lipid transport and metabolism
P	63	2.09	Inorganic ion transport and metabolism
Q	30	0.99	Secondary metabolites biosynthesis, transport and catabolism
R	0	0.00	General function prediction only
S	569	18.87	Function unknown
Multi COG	59	1.96	Multiple COG assignments
Single COG	1,963	65.09	single COG assignments
No COG	994	32.96	Not in COGs

**Figure 2:**
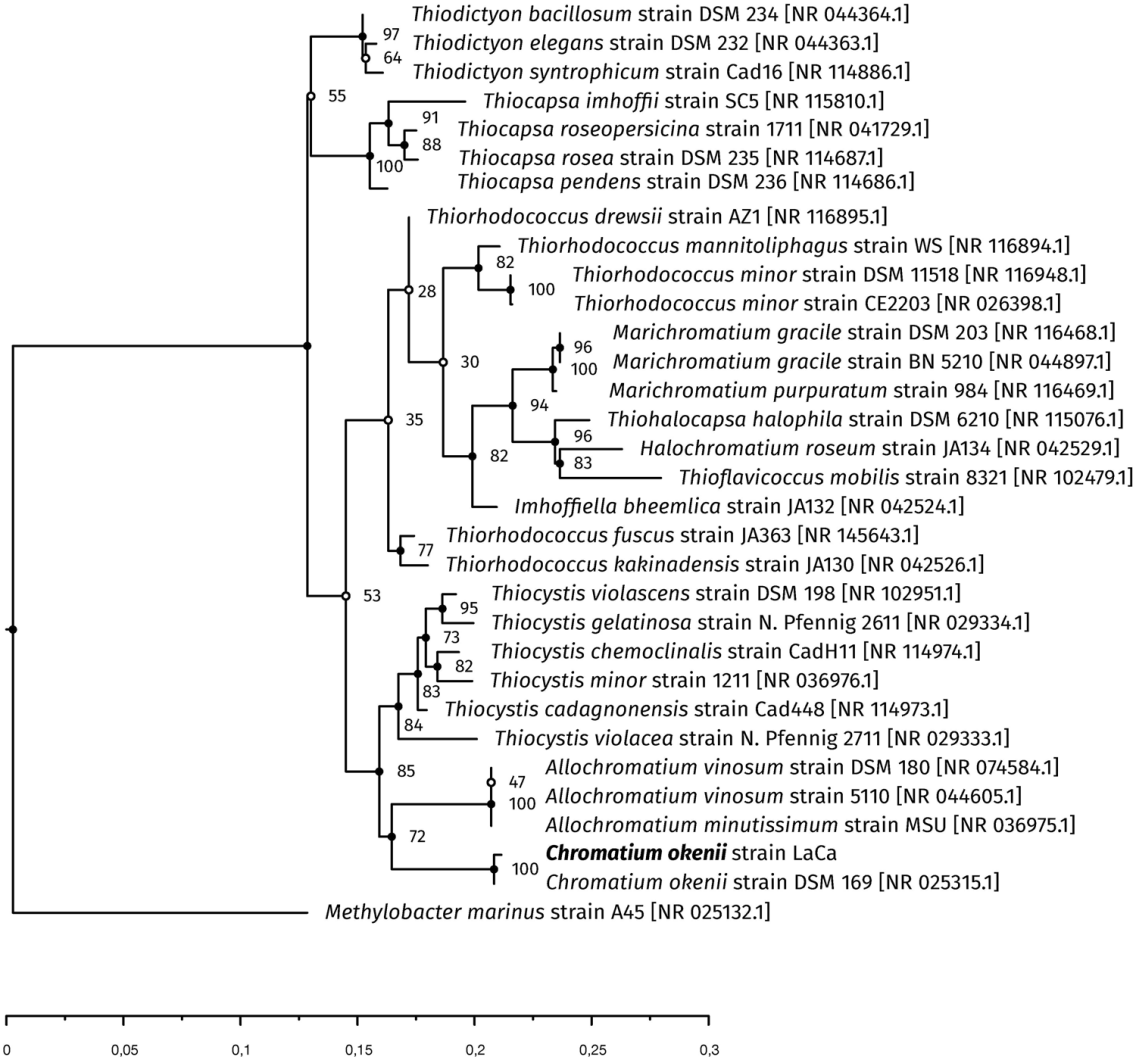
Phylogenetic relationship of *Chromatium okenii* strain LaCa based on 16S rRNA gene sequences. IQTree^49^ was used to calculate a consensus tree combining 1,000 bootstrap iterations and 1,000 aLRT replications. Branch lengths were optimized by maximum likelihood on original alignment. Full node circles indicate bootstrap support above 60%. Scale bar denotes genomic distance in base-pair (bp) substitutions per 100 bp.

Phylogeny based on the 16S rRNA gene revealed a 99% sequence identity with *C*. *okenii* DSM 169^T^ and *C*. *okenii* strain LaCa groups with *Allochromatium* and *Thiocystis* spp. (Fig. 2). When comparing a subset of 100 core genes of sequenced Chromatiaceae, *C*. *okenii* is closely related to *T*. *violascens* and *A*. *vinosum* (Supplementary Fig. S4). Since the *C*. *okenii* DSM 169^T^ type strain or other *Chromatium* spp. are not available anymore in strain collections^54^, a more detailed genomic comparison within the genus *Chromatium* was not possible.

### Genome Features

**Figure 3:**
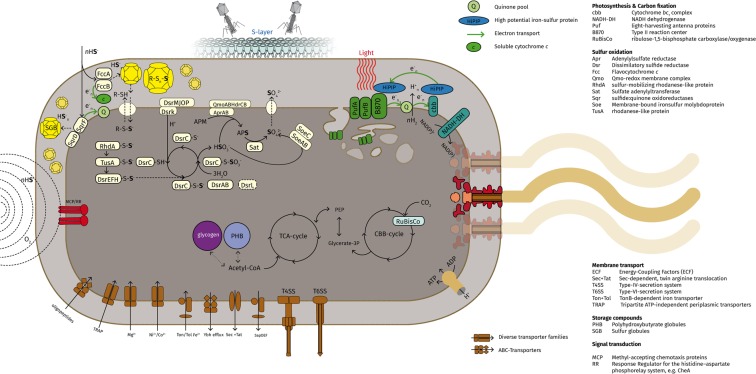
Schematic *Chromatium okenii* cell with the discussed metabolic and structural features indicated. (Phage scheme adapted from “*PhageExterior*.*svg*” by “*Adenosine*”, used under the ‘Creative Commons’ Attribution - Share Alike 3.0 Unported license).

An overview of the features discussed below is depicted in a cell scheme in Fig. 3.

### Photosynthesis and Chemotrophy

For *C*. *okenii*, an extensive system of photosynthetic membranes has been characterized^55^ where type II reaction centres (RCs) are used to transform light energy into chemical energy. In the strain LaCa genome, a canonical PSB photosystem II-type RC is encoded in two clusters, containing two *pufAB* (CXB77_RS07530, CXB77_RS07535, CXB77_RS07560 and CXB77_RS07565), *pufL* (CXB77_RS07555) and *pufM* (CXB77_RS07550) genes, a RC complex subunit H *puhA* (CXB77_RS09135) and a putative photosynthetic complex assembly protein (CXB77_RS09130). Additional light harvesting complex LHC I and LHC II genes (CXB77_RS02170 and CXB77_RS02175) and two extra pairs of *pufAB* genes were identified (CXB77_RS10755–CXB77_RS10765). In accordance with findings in other PSB, multiple gene copies of LHC are thought to be an adaptation to changes in light availability^56^ and were also found in “*Thiodictyon syntrophicum*” strain Cad16^T^ and *A*. *vinosum* DSM180^T^ ^57^. For *C*. *okenii*, low light adaptation was elucidated by measuring fluorescence kinetics *in situ*^58^ and quantum yields below the optimum were observed^59^. In PSB, light is taken up efficiently by photosynthetic pigments and the energy obtained is then further transferred to the reaction centers (RCs). Both, the carotenoid okenone^60^ and BChl *a*^14^ are synthesized in *C*. *okenii* strain LaCa. In agreement, the complete genes encoding for BChl *a* synthesis (CXB77_RS09140–CXB77_RS09180) and of the carotenoid okenone (*crt* and *cru*) were found. Notably, a carotenoid 3,4-desaturase *crtD*-homologue of the C-4/4′ ketolase *cruO-*type (CXB77_RS02160)^61^ was detected in proximity to the *crtC* hydroxyneurosporene synthase gene (CXB77_RS02155) homologous as in *Marichromatium purpuratum* DSM 1591^T^. Interestingly, *C*. *okenii* strain LaCa showed a stable BChl *a* to protein ratio over a three months sampling period *in situ*^23^. The BChl *a* synthesis rate in the dark was thereby found to be independent of sampling depth. However, subtle changes in light intensity (0.06 mol quanta m^−2^ h^−1^ in average) had a significant impact on the successive BChl *a* synthesis rates^23^. In summary, *okenii* strain LaCa may modulate light dependent energy uptake efficiency by different combinations of LHC antenna proteins and pigments concentrations, respectively.

Soluble electron-carrier cytochromes ensure cyclic electron flow by shuttling electrons from the cytochrome *bc*1 complex back to the RCs during photosynthesis. In strain LaCa, the high potential iron sulfur protein (HiPIP; CXB77_RS02565) possibly function as the key high potential cytochrome as in *A*. *vinosum* DSM180^T^ ^61^. Furthermore, cytochrome *c*551/*c*552 (CXB77_RS07885) may serve as an extra RC reductant under autotrophy^62^. Variable soluble electron carriers found were cytochrome *c*′ (CXB77_RS15975), cytochrome *c*4 (CXB77_RS04500) homologous as in *Thiocapsa roseopersicina* and two soluble *c*-type cytochromes (CXB77_RS01960–CXB77_RS01970 and CXB77_04510) most closely related to the gene in *A*. *vinosum* DSM180^T^.

In stratified lakes, motile *Chromatium* spp. have been found in viable, non-dividing states below the chemocline^62^ and survival over 1.5 year in darkness has also been described^63^. In the chemocline of Lake Cadagno, the greatly diminished numbers during winter is possibly due to the low light availability of >0.4 *µ*mol quanta·m ^−2^ s^−1^ ^21^. Interestingly however, upward motility of *C*. *okenii* in dark conditions in Lake Cadagno has been inferred indirectly by cells at the underside of sediment traps in spring^24^ and by detection of nocturnal bioconvection in summer^31^. Both findings may point to the importance of dark heterotrophic metabolism for *C*. *okenii* vitality. A relatively low oxycline has been observed from October to December^29^ due to thermal mixing of the mixolimnion. Furthermore, oxygen (<20 nmol L^−1^) produced *in situ* by oxygenic photosynthesis has been detected in summer^64^. Together, these observations infer the possibility of micro-oxic conditions at the chemocline throughout most time of the year. Despite that aerobic sulfur oxidation yields only about 25–30% of the energy provided by anaerobic photosynthesis^65^, this amount may still be critical for persistence of these microorganisms and the mixotrophic growth in summer.

Accordingly, a complete respiratory chain was found in *C*. *okenii* strain LaCa including NADH-quinone oxido-reductase (CXB77_RS02240–CXB77_RS02300 and CXB77_RS02310), succinate-dehydrogenase (CXB77_RS02325–CXB77_RS02335 and CXB77_RS09655), as well as a multi-subunit terminal cytochrome *bd* oxidase (CXB77_RS12155 and putatively CXB77_RS12145 and CBX77_RS12150). Both can function as terminal electron acceptor in photosynthesis and substrate respiration. Taken together, we found evidence on the genomic level that *C*. *okenii* strain LaCa may be able to perform anoxygenic photosynthesis and chemotrophic respiration in Lake Cadagno, as generally suggested for PSB by Kämpf and Pfennig^66^. Interestingly, partly complementary information to our findings is given by a metagenomic sequence bin in Berg *et al*.^33^.

In contrast, no significant growth after five days in darkness was shown under chemotrophic incubations at room temperature with two strains of *C*. *okenii* under a 5% O_2_ atmosphere^66^. Additionally, no correlation between light availability and specific dark fixation rates was observed *in situ* in a chemocline population dominated by *C*. *okenii*^59^. Consequently, the combination of the experimentally described metabolism and the biological functions encoded in the genome of *C*. *okenii* does not readily explain the high dark total fixation rates measured in Lake Cadagno^28,67^. Interestingly however, *C okenii* might be able to combine aerobic and anaerobic carbon fixation pathways by actively moving along the vertical oxygen gradient in summer conditions^33^.

### Sulfur Metabolism

*C*. *okenii* uses reduced sulfur compounds such as H_2_S and S^0^ as reductants for photolitho-autotrophic growth^68,69^. Subsequently, light energy is used to transfer electrons to NAD(P)^+^ and ferredoxin for CO_2_ fixation. In accordance, *C*. *okenii* strain LaCa encodes flavocytochrome *c* (FccAB; CXB77_RS06380) and the sulfide:quinone oxidoreductases (SqrD and SqrF; CXB77_RS06755 and CXB77_RS12425) that both oxidize H_2_S in the periplasm to form sulfur globules (SGBs) containing S^0^. The SGBs are surrounded by sulfur globule proteins (SGPs) that fold into collagen-like filaments^70^ filaments. Accordingly, we identified two putative SgpA copies with N-terminal signal peptides (CXB77_RS07855 and CXB77_RS14820). This is important, as the homologue SgpA is essential to build intact sulfur globules in *A*. *vinosum*^71^. Furthermore, the canonical dissimilatory sulfite oxidation pathway (Dsr) that enables sulfite production in the cytoplasm in PSB^72^ was found completely conserved in *C*. *okenii* strain LaCa in one cluster (CXB77_RS03215–CXB77_RS03270) and shows gene synteny to other Chromatiaceae. Interestingly, two *arsR* family transcriptional regulator genes (CXB77_RS06260 and CXB77_RS07240) possibly involved in H_2_S-dependent gene regulation^73^ were also detected. Moreover, in strain LaCa we found the trimeric adenylylsulfate reductase alpha and beta-subunits AprAB (CXB77_RS17245 and CXB77_RS17240) that is anchored by the CoB–CoM heterodisulfide reductase multi subunit complex (CXB77_RS04305–CXB77_RS04320). To complete sulfur oxidation, a Sat sulfate adenylyltransferase (CXB77_RS09675) and the dissimilatory-type SoeABC type enzyme (CXB77_RS11845–CXB77_RS11855) are encoded. An additional cluster of sulfur carrier proteins TusA (XB77_RS15940) and DsrE2 (CXB77_RS15945)^74^ putatively involved in sulfur oxidation were detected. Furthermore, we identified a cytochrome *b*561 (CXB77_RS01235) and an octaheme cytochrome *c* (CXB77_RS01240) homologues to *A*. *vinosum* DSM180^T^. Both enzymes are conserved among PSB and have been found to be upregulated in *A*. *vinosum* DSM180^T^ with sulfide as sole electron donor^75^. Notably, no genes encoding Sox proteins necessary for thiosulfate (S_2_O_3_^2−^) oxidation were found^76^, which is in accordance with previous experimental results^69^. Furthermore, no genes of the adenylyl-sulfate kinase Cys-pathway for assimilatory sulfate reduction could be detected, confirming previous experimental findings where sulfate uptake was not observed for *C*. *okenii*^69^. Finally, for *C*. *okenii* no hydrogenases were predicted in the genome that excludes H_2_ as a source of electrons^68,69^.

### Nitrogen and Phosphate Assimilation

We detected *nif* genes involved in nitrogen fixation in the *C*. *okenii* strain LaCa genome distributed throughout the genome as in *A*. *vinosum* DSM180^T^ ^77^. The presence of a dimeric nitrogenase molybdenum-iron protein *nifDK* (CXB77_RS12525 and CXB77_RS12530) and the nitrogenase iron protein *nifH* (CXB77_RS12535) point to a diazotrophic metabolism. Additionally, homologues *nifD* sequences were identified in *T*. *violascens*, *Lamprocystis* spp. and “*T*. *syntrophicum*”. N_2_-uptake is under transcriptional control of the two-component sensor histidine kinases NtrX and NtrY (CXB77_RS03520/ CXB77_RS03525), the nitrogen regulatory protein P-II (CXB77_RS11185) and *nifA* (CXB77_RS10450), as well as the oxygen sensor *nifL* (CXB77_RS10445). In strain LaCa, we found polyphosphate kinase and exopolyphosphatase encoded, however previous studies failed to demonstrate *in situ* polyphosphate accumulation^78^. Furthermore *C*. *okenii* strain LaCa also encodes genes for ammonium assimilation, glutamate synthase and glutamine synthetase. In accordance, *in situ* NH_4_^+^-consumption of *C*. *okenii* was demonstrated^27^ and modelled for the Lake Cadagno chemocline^25^. However, alternative N-uptake mechanisms must have still to be described.

### Carbon Metabolism

The light driven carbon uptake kinetic has been studied in detail in *C*. *okenii* before^69,79^. In PSB, the Calvin-Benson-Bassham-cycle (CBB) is the central carbon assimilation mechanism^69,79,80^. For the genome of strain LaCa a complete CBB cycle with the one *cbbM* ribulose 1,5-biphosphate carboxylase/oxygenase (RuBisCO) form II (CXB77_RS09535), two regulatory genes *cbbQ* (CXB77_RS09540) and *cbbO* (CXB77_RS09550), and phosphoribulokinase PrkB (CXB77_RS15420) were described. Furthermore, an additional RuBisCO-like protein gene *rbcL* (CXB77_RS07520) is also present in the genome. The RbcL is putatively involved in methionine salvage and it is typically found in purple bacteria, such as *Rhodopseudomonas palustris*^81,82^. Alternatively, RbcL also functions in a stress response mechanism or in sulfur metabolism in the GSB *Chlorobium tepidum*^83^ and the purple bacteria *Rhodospirillum rubrum*^84^ or in stress response. In the genome of strain LaCa, no hypothetical carboxysome-like subunits and RuBisCO form I (*cbbL* and *cbbS*) were found. This is in contrast to other small-celled PSB such as “*T*. *syntrophicum*” strain Cad16^T^ and *Lamprocystis* spp. isolated from Lake Cadagno^85,86^.

For PSB, several carbon storage mechanism have been described^87,88^ that possibly serve both as energy and reductant reserves. In *C*. *okenii* strain LaCa, glycogen storage is mediated through glucose-1-phosphate adenylyltransferase and a 1,4-alpha-glucan (glycogen) branching enzyme (CXB77_RS16905). Furthermore, we found a complete tricarboxylic acid (TCA) cycle and enzymes for glycolysis. For *C*. *okenii*, polyhydroxybutyrate (PHB) synthesis under nitrogen limitation was described *in vitro*^78^ and a high average C:N ratio of 14.8 was previously reported that could potentially could induce carbon storage mechanisms. In accordance, genes encoding PhaC (CXB77_RS16475) and PhaE (CXB77_RS16480) involved in PHB synthesis and depolymerisation are present in *C*. *okenii* strain LaCa. Furthermore, *C*. *okenii* possibly oxidizes glycogen to PHB and stored sulfur is used as an electron sink by reduction into H_2_S^79^. Additionally, for *C*. *okenii* under *in situ* dark conditions the putatively aerobic oxidation of sulfur was found to be favoured over glucose oxidation to acetate and CO_2_^78^ under *in situ* dark conditions. However, PHB inclusions were not observed and storage compounds were depleted within hours under *in situ* conditions in Lake Cadagno^78^. These results obscure the role of PHB for long time survival of *C*. *okenii*. For the small-celled PSB “*T*. *syntrophicum*” strain Cad16^T^, proteins involved in the degradation of PHB were shown to be upregulated in the dark conditions *in vitro*^89^ and under micro-oxic conditions *in situ*^90^. The degradation of PHB granules results in acetyl-CoA and NAD(P)H, which are both needed in the CO_2_ fixing process in the absence of light.

### Membrane Transport and Bacterial S-layer

Similar to other PSB species such as *A*. *vinosum* DSM 180^T^ or “*T*. *synthrophicum*” strain Cad16^T^, the genome of *C*. *okenii* strain LaCa encodes both a Type IV pilus (CXB77_RS13225, CXB77_RS13640–CXB77_RS13660 and CXB77_RS13745–CXB77_RS13760) and a Type VI secretion system (CXB77_RS0616–CXB77_RS06190 and CXB77_RS12705–CXB77_RS12755). Other secretion systems encoded are a general secretion (Sec) and twin-arginine translocation (Tat). Moreover, we found ABC-type transporters for di-peptide, oligopeptide, lipoprotein, phosphate and molybdenum uptake, as well as Tol and TRAP and Co^2+^, Mg^2+^ and Ni^2+^-uptake systems.

The main function of the surface layer (S-layer) is to reinforce bacterial cells against osmotic, mechanical and thermal forces^91^. Moreover, the S-layer possibly also functions as protection against bacterial predation and bacteriophage infection^91,92^. The S-layer in *C*. *okenii* consists of conical shaped hexagonal lattice subunits with a diameter of 13 nm that are regularly spaced by 19 nm and extend 25 nm from the surface^54^. Accordingly, two putative exported S-layer proteins (CXB77_RS09990 and CXB77_RS09995) and a FhaB-like protein (CXB77_RS10005) similar to alkaline phosphatases in *Microcystis* spp. were identified in strain LaCa. The S-layer proteins might be exported through a homologues Type I secretion SapDEF system (CXB77_RS09940–CXB77_RS09950)^88^. We also found a putative SapC protein (CXB77_RS08915), missing the signal peptide homologous to *Halorhodospira halochloris* (HH1059_1773).

In Lake Cadagno the *C*. *okenii* population was observed to wither dramatically within a period of days in October^21,22^. The increase in *C*. *okenii* cells over the preceding summer months possibly leads to metabolic stress and an increased sedimentation rate could lead to conditions of high bacterial predator pressure. In accordance, epibionts were reported for *C*. *okenii* in Lake Cadagno^27^ and for other large-celled *Chromatium* species elsewhere^93^. These were characterized as bacterial scavengers that feed on non-dividing *Chromatium* cells^94,95^ and may lead to this population collapse^96^. In contrast, no sequences related to *Bdellovibrio*, *Daptobacter* or *Vampirococcus*-type could be detected in the enrichment samples. While this data is currently unavailable, we expect to detect epibionts on non-viable, sedimented *C*. *okenii* cells in samples from the lower monimolimnion.

The importance of bacteriophages for aquatic microbial community dynamics has been recognized^97,98^ however few studies have focused on stratified systems^99–101^. In this study, several putative prophage and incomplete phage sequences were found in the *C*. *okenii* strain LaCa sequence (Supplementary Table S5 and Supplementary Fig. S5). The prophage sequences were thereby similar to sequences from *T*. *violascens* DSM 198 and *A*. *vinosum* DSM 180 and the putative phage Chok4 showed sequence similarity to *T*. *violascens* DSM 198 and *A*. *vinosum* DSM 180 using *BLASTn*. When the database was restricted to viral sequences no significant hits were obtained. Furthermore, a 442 bp CRISPR with seven 36 bp repeats and six spacers was detected on contig PPGH01000029.1 (Supplementary Table S6). A similar CRISPR-sequence was found in *A*. *vinosum* DSM180^T^ on plasmid pALVIN01. However, no adjacent CAS protein cluster was detected in the genome of *C*. *okenii* strain LaCa. Furthermore, eleven Rha-type phage regulatory proteins were detected. In summary, putative phage sequences and phage-related genes found indicate the presence of phages specific for the dense chemocline community. Therefore, the function of the S-layer against phage attachment, as well as the Type 6 secretion system in defence against bacterial predation, must be further elucidated.

### Flagella and Chemotaxis

*C*. *okenii* is motile using around 40 lophotrichous flagella that together form a tuft with a length of 20 to 30 *µ*m^6,54^. The direction is controlled by the action of either pulling or pushing flagella that rotate clockwise or counter clockwise, respectively^99^. In accordance, one cluster with genes encoding the basal body, hook and filament were identified. Additional genes *flg*, *flh*, *che* and *fli* were identified to group with *motA* and *motB* genes. A histidine–aspartate phosphorelay (HAP) based system^100^ that comprises chemotaxis genes *cheABRWYZ* and in total 31 putative chemoreceptors (MCP: methyl-accepting chemotaxis protein) of the TAP or TLPA family were detected. Notably, a putative Aer aerotaxis sensor receptor protein (CXB77_RS12890), bacteriophytochrome (CXB77_RS05740) and two putative blue-light-activated histidine kinases (CXB77_RS09475 and CXB77_RS08785) were found. We also detected a putative circadian input kinase *A* (CXB77_RS08775), however no complete *kaiABC* relay was identified. Interestingly, only parts of a set of genes involved in acyl homoserine lactone mediated quorum sensing were detected, including components of the SagS-HptB-HsbR (swarming activity and biofilm formation) two-component regulatory system/cAMP/Vfr signalling (CXB77_RS11060 and CXB77_RS08790) and the putative transcriptional activator protein LasR (CXB77_RS11545).

Large- celled *Chromatium* spp. have been characterized as metabolically less flexible in comparison with the non-motile, small-celled PSB^56,68^ and might therefore be forced to adapt to changing conditions by constantly moving along the optimal gradients. Overmann and Pichel-Garcia postulate that motile PSB have an advantage over PSB with gas vacuoles at light intensities above 0.2 *µ*mol quanta·m ^−2^ s^−1^ ^56^. In Lake Cadagno, between 5.8*–*35 *µ*mol quanta m^−2^ s^−1^ were measured at the upper chemocline during summer, whereas a ten-fold decrease in light intensity within the cm to m thick bacterial layer was found^17^. Moreover, an inverse correlation between available light and thickness of the bacterial plume was described for Lake Cadagno^23^. That may indicate that members of the microbial population actively move vertically on a minute to hour timescale. In comparison, the velocity of *Chromatium minus* seems to be determined defined by external sulfide concentration and light intensity *in vitro*^102^. Interestingly, for *C*. *minus* both swimming speed and run time, respectively, are higher and longer under low light intensity when compared to high light conditions, a phenomena that persists over hours^102^. The observed swimming speed of (2.7 ± 1.4) × 10^−5^ m s^−1^ ^32,103^ (0.97 m h^−1^) of strain LaCa enables the vertical crossing of the chemocline the chemocline can be crossed within minutes to hours. Importantly, the resulting accumulations of motile, dense cells at the upper border of the chemocline may provoke bioconvection^32^. Taken together, these different observations indicate that *C*. *okenii* would benefit from upward movement under non light-limiting conditions under the guidance of scotophobotaxis, negative O_2_ and positive H_2_S chemotaxis, respectively. However, temporal vertical mobility patterns of *C*. *okenii* have been additionally described as diel^10,104,105^ or stochastic^31,32^. The intervened signalling pathways that coordinate movement in *C*. *okenii* will also have to be examined in more detail.

### Comparative genomics

**Figure 4:**
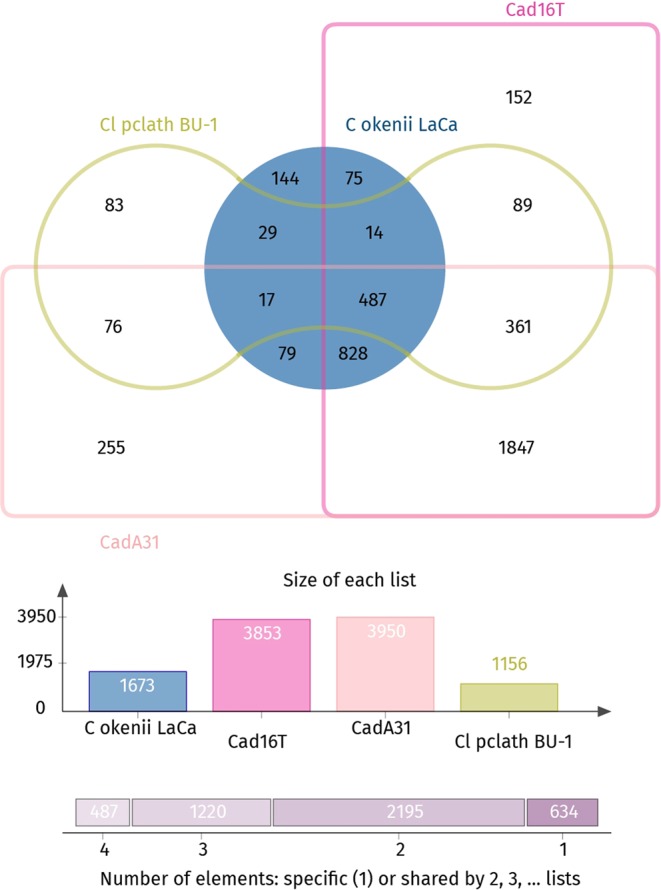
Venn diagram showing the shared orthologous gene clusters among four sulfur oxidizing bacteria found in Lake Cadagno. Denoted the number of clusters (orthologues or paralogs) shared between phototrophic sulphur bacteria species. Each cluster contains at least two genes.

Orthologue gene families can be used to compare the encoded metabolic, structural and behavioural potential between organisms^106^ and have here been applied in the study of encoded differences between PSB and GSB. When we compared the KEGG enzymatic pathways between PSB only minor differences were detected (Supplementary Table S7). Subsequently, *OrthoVenn* was used to create a dataset of annotated gene clusters to compare phototrophic sulfur bacteria population of Lake Cadagno. Thereby, the genomes of previously isolated PSB (“*T*. *syntrophicum”* strain Cad16^T^ and *L*. *purpurea* strain CadA31) and GSB (*C*. *phaeoclathratiforme* strain Bu-1) were compared to PSB *C*. *okenii* strain LaCa. Using an *in silico* approach, we sought to find genes potentially elucidating the co-existence of an oxygenic phototrophic sulfur bacteria in the chemocline. In total, 10,632 genes were included, and the four species encompassed 4,536 gene clusters, 3,902 orthologous gene clusters –at least containing two species– and 386 single-copy gene clusters (Fig. 4).

Orthologous gene clusters shared by PSB (*n* = 828) were enriched for GO-terms protein export and membrane insertion, as well as light harvesting complex components and cyclic electron flow, indicating the primary phototrophic lifestyle and possibly the membrane bound enzymes (e.g. RCs) involved. Whereas GSB *C*. *phaeoclathratiforme* strain Bu-1 was enriched for chlorosome components among others, “*T*. *syntrophicum*” strain Cad16^T^ was enriched for chitinase function and extracellular and outer membrane components and *L*. *purpurea* strain CadA31 for phage related sequences and processes, respectively.

Conserved RuBisCO type II (CbbM) and RuBisCO-like (RPL) type IV sequences were detected in all PSB examined here. Interestingly, the heterodimeric RuBisCO type I (CbbLS) is missing in *C*. *okenii* and was found only in the both small-celled PSB. Additionally, all PSB studied encoded cytochrome *d* ubiquinol oxidases (CydAB), whereas only small-celled PSB encoded a *ccb*3 type cytochrome *c* oxidase (Table 3).

**Table 3:** Genome features and growth characteristics of *Chromatium C*. *okenii* strain LaCa and of selected purple sulphur bacteria. CBB: Calvin-Benson-Bassham cycle.

Genome features	“*C*. *okenii*” str. LaCa	“ *T*. *syntrophicum*” str. Cad16^T^	*L*. *purpurea* str. CadA31*	*T*. *violascens* DSM 198^T^	*A*. *vinosum* DSM 180^T^
Genome size [Mb]	3.78	7.74	7.19	5.02	3.67
Number of scaffolds	1	1	302	1	1
Number of contigs	45	3	302	1	3
Average G + C content [%]	49.8	66.2	64.3	62.6	64.2
Number of genomic objects (CDS, fragment CDS, r/tRNA)	3,792	6,601	7,314	4,555	3,366
Number of coding sequences (CDS)	3,016	6,237	7,255	4,317	3,302
Motility	+	−	−	+	+
Carbon fixation	CBB	CBB	CBB	CBB	CBB
Thiosufate oxidation	−	+	+	+	+
Chemotrophic growth	−	+	+	+	+
Hydrogenases	−	+	+	+	+
Catalases	−	+	+	+	+
cbb 3 type terminal cytochrome *c* oxidase	−	+	+	+	+
Pigments	BChl *a*, okenone	BChl *a*, okenone	BChl *a*, okenone	BChl *a*, rhodopinal	BChl *a*, spirilloxanthin
Vitamin B12 requirement	+	+	+	+	+
Generation time [h]	120–168	121	33–35	NA	13–20

In *C*. *okenii*, out of 3,016 protein coding genes, 144 exclusive gene-clusters were present. GO-enrichment analysis within this group resulted in over-representation of GO-terms linked to chemotaxis, flagellar movement, the S-layer and arginine uptake, respectively (Fig. 4). Arginine uptake may be important for *C okenii*, since microbial utilization of free amino acids in lakes has been described as a driver for bacterial community function^107^ and arginine ammonification has also been used as a proxy for respiration of microbial communities^108,109^. For strain LaCa, arginine could therefore function as an additional carbon source not available to other PSB and GSB, and also provide extra N due to the high C:N ratio of 3, as proposed for other freshwater bacteria^109^.

*C*. *okenii* comprises an approximately 7× larger cell volume^27^ and a 30× reduced surface-to-volume ratio compared to small-celled PSB. As bacterial cell size influences metabolic activity and internal organization^110,111^, transcriptional regulation, functional compartmentalisation and genome organisation (i.e. polyploidy) may be fundamentally diverse between *C*. *okenii* and small-celled PSB and GSB. However, no evidence of multiple chromosomes in *C*. *okenii* strain LaCa was found when taking into account the uniform coverage and the lack of allele variants of the assembly, respectively.

## Conclusions

In the study presented, we could we confirmed several previous experimental findings of metabolic activity^66,68,78,79^ on the basis of the genomic information. Typically for PSB, the *C*. *okenii* strain LaCa genome encodes the CBB-cycle and a type II RC, however *sox*-proteins, hydrogenases and the Cys sulfate assimilation pathway are missing completely. Furthermore, genes involved in carbon and nitrogen utilization were similar between *C*. *okenii* strain LaCa and other PSB and show redundancy with *A*. *vinosum* DSM 180^T^. Interestingly, cytochrome *d* ubiquinol oxidases were also found in all known PSB genomes of Lake Cadagno, indicating aerobic respiration of oxidized organic carbon compounds, such as glucose. In contrast, the co-occurrence of RuBisCO type II together with *cbbQ* and *cbbO* genes as in *C okenii* has been described in more detail for obligate autotrophs^112^. However, in the PSB “*T*. *syntrophicum*” strain Cad16^T^ the type II RuBisCO was constitutively expressed at dark and light conditions^89,90^ and it was therefore suggested to be function in cofactor re-oxidation as it was found in in purple non sulfur bacteria^81^. Taken together, the absence of both, a type I RuBisCO (CbbLS) and a carboxysome-like CO_2_ concentration mechanism in *C*. *okenii*, as well as known low CO_2_ affinity of RuBisCO form II^113^, possibly inhibits the cell functioning at low surrounding CO_2_ concentrations.

In terms of changes in the environment, the *C*. *okenii* population in the Lake Cadagno is exposed to abiotic factors that vary on the short-term (minutes to hours), such as light availability, reduced electron donors and oxygen, disturbances of the water column (i.e. internal waves and seiches) and biotic factors, such as grazing pressure^67,114^. Seasonal factors such as an increase in total cell numbers within the chemocline in summer, changes in the day to night length ratio and the 3–5 months of ice-cover in winter –i.e. light availability and quality– add up additional complexity.

Under low light availability in spring, the reported relative higher sulfide affinity in comparison with other PSB and the benefit of the larger dark-to-light hours ratio may, in turn, give *C*. *okenii* an advantage over small celled PSB as observed *in vitro*^115^. Furthermore, the low phototrophic population cell concentration of ~25% of the summer community^21^ may also reduce self-shading^116^ and predation rates and phage numbers may also be lower. The rapid onset of aerobic photosynthesis after the ice-melt may additionally support chemotrophic microaerophilic growth of *C*. *okenii*.

To conclude, the multiple factors that influence *C*. *okenii* strain LaCa behaviour have to be further disentangled. The sensing of short-term fluctuations and adaptation to more dramatic longer-lasting changes in the environment were found to have left an imprint in the *C*. *okenii* genome by the relative over-representation of genes for motility and sensing, and in some versatility in the assimilatory pathways. Chemo- and scotophobotaxis, quorum sensing and diel and seasonal behavioural patterns have must be considered in future studies on bioconvection. Further studies on genomic heterogeneity within the *C*. *okenii* population or, and diversity transcriptional control on single cell level could give further insight on the important ecological role of *C*. *okenii* for the Lake Cadagno ecosystem^22,27^ and other stratified water bodies.

## Supplementary information

Supplementary Information

## Data Availability

The complete, corrected and annotated genomic data is available at NCBI under the GenBank assembly accession: GCA_002958735.1 and RefSeq assembly accession: GCF_002958735.1.
