# Supplementary material for: Draft Genome Sequence of Chromatium okenii Isolated from the Stratified Alpine Lake Cadagno
Source: Sci Rep. 2019 Feb 13;9:1936. doi: 10.1038/s41598-018-38202-1 (PMC6374484; doi:10.1038/s41598-018-38202-1)
Supplement: Supplementary file 1 — Supplementary Information [file 41598_2018_38202_MOESM1_ESM.pdf]

Supplementary Material:

Draft Genome Sequence of *Chromatium okenii* Isolated from the Stratified Alpine Lake Cadagno

Samuel M Luedin [1 2 3*], Nicole Liechti [3 4 5], Raymond P Cox [6], Francesco Danza [1 2], Niels-Ulrik Frigaard [7], Nicole R. Posth [8 9], Joël F Pothier [10], Samuele Roman [2 11], Nicola Storelli [2], Matthias Wittwer [3] and Mauro Tonolla [1 2 11‡]

1 Microbiology Unit, Department of Botany and Plant Biology, University of Geneva, Geneva, Switzerland

2 Laboratory of Applied Microbiology, Department of Environment, Constructions and Design, University of Applied Sciences of Southern Switzerland (SUPSI), Bellinzona, Switzerland

3 Biology Division, Spiez Laboratory, Federal Office for Civil Protection, Spiez, Switzerland

4 Interfaculty Bioinformatics Unit, University of Bern, Bern, Switzerland

5 Graduate School for Cellular and Biomedical Sciences, University of Bern, Bern, Switzerland

6 Department of Biochemistry and Molecular Biology, University of Southern Denmark, Odense, Denmark

7 Department of Biology, University of Copenhagen, Helsingør, Denmark

8 Department of Biology, University of Southern Denmark, Odense, Denmark

9 Current: Department of Geosciences and Natural Resource Management (IGN), University of Copenhagen, Copenhagen, Denmark

10 Environmental Genomics and System Biology Research Group, Zurich University of Applied Sciences (ZHAW), Wädenswil, Switzerland

11 Alpine Biology Center Foundation, Bellinzona, Switzerland

Corresponding Authors

***Samuel Luedin
samuel.ludin@etu.unige.ch

‡Prof. Mauro Tonolla
mauro.tonolla@supsi.ch

Supplementary Tables

Supplementary Table S 1 | Classification and general features of *Chromatium okenii* str. LaCa according to the MIGS recommendations [1]

MIGS ID	Property	Term	Evidence code[a]
	Classification	Domain *Bacteria*	TAS [2,3]
		Phylum *Proteobacteria*	TAS [2]
		Class *Gammaproteobacteria*	TAS [2]
		Order *Chromatiales*	TAS [2]
		Family *Chromatiaceae*	TAS [2]
		Genus *Chromatium*	TAS [2,4]
		Species *okenii*	TAS [4,2]
		Strain: LaCa	NAS
	Gram stain	Negative	TAS [6]
	Cell shape	Rod	TAS [4]
	Motility	Motile	TAS [5]
	Sporulation	No	NAS
	Temperature range	Not determined	NAS
	Optimum temperature	20–35	TAS [6]
	pH range; Optimum	Not determined	NAS
	Carbon source	CO_2, acetate	TAS [7]
MIGS-6	Habitat	Fresh water, alpine meromictic lake	TAS [6,8]
MIGS-6.3	Salinity	Not determined	NAS
MIGS-22	Oxygen requirement	No	TAS [7]
MIGS-15	Biotic relationship	Free-living	TAS [6,8]
MIGS-14	Pathogenicity	Non-pathogen	NAS
MIGS-4	Geographic location	Switzerland, Ticino	TAS [8]
MIGS-5	Sample collection	14 July 2016	NAS
MIGS-4.1	Latitude	46°33′ N	TAS [8]
MIGS-4.2	Longitude	8°43′ E	TAS [8]
MIGS-4.4	Altitude	1,923 m	TAS [8]

[a]Evidence codes – *IDA* Inferred from Direct Assay, *TAS* Traceable Author Statement (i.e., a direct report exists in the literature), *NAS* Non-traceable Author Statement (i.e., not directly observed for the living, isolated sample, but based on a generally accepted property for the species, or anecdotal evidence). These evidence codes are from the Gene Ontology project [9]

Supplementary Table S 2 | Genome completeness estimation for the *Chromatium okenii* str. LaCa using CheckM v1.2.2. *CheckM* [10] was used to infer genome completeness, contamination and heterogeneity of the *C. okenii* str. LaCa enrichment. The Taxonomic-specific Workflow [11] was set to *Chromatiaceae* and the other parameters were set to default.

Contigs	Marker lineage	# genomes	# markers	# marker sets	0	1	2	3	4	5+	Com-plete-ness (%)	Contami-nation (%)	Strain heterogeneity (%)
45 contigs, canu v1.4	Chromatiaceae (4)	14	545	300	55	434	54	1	1	0	88.89	11.42	95.24
8 longest contigs, canu v1.4	Chromatiaceae (4)	14	545	300	56	481	8	0	0	0	88.64	1.11	87.5

Supplementary Table S3 | List of multiple occurring *Chromatiaceae* lineage specific marker genes in the *Chromatium okenii* str. LaCa genome. *CheckM* [10] was used to infer the 56 multiple marker genes within the 45 contigs

Bin Id	Marker Id	Gene Ids
Cokenii.contigs.canu	TIGR00761	tig00000032_5,tig00000296_696
Cokenii.contigs.canu	TIGR00442	tig00000029_4,tig00000296_271
Cokenii.contigs.canu	PF00162.14	tig00000031_55,tig00000112_10,tig00000165_29
Cokenii.contigs.canu	PF01018.17	tig00000031_81,tig00000088_325
Cokenii.contigs.canu	PF00490.16	tig00000021_15,tig00000296_542
Cokenii.contigs.canu	PF01386.14	tig00000016_2,tig00000296_53
Cokenii.contigs.canu	PF01016.14	tig00000031_82,tig00000088_324
Cokenii.contigs.canu	PF06508.8	tig00000036_15,tig00000296_949
Cokenii.contigs.canu	PF00334.14	tig00000029_8,tig00000296_275
Cokenii.contigs.canu	TIGR00195	tig00000032_1,tig00000296_693
Cokenii.contigs.canu	PF07517.9	tig00000031_86,tig00000088_320
Cokenii.contigs.canu	PF02075.12	tig00000017_14,tig00000296_211
Cokenii.contigs.canu	TIGR03300	tig00000029_2,tig00000296_269
Cokenii.contigs.canu	TIGR00878	tig00000022_2,tig00000296_453
Cokenii.contigs.canu	PF01043.15	tig00000031_86,tig00000088_320

Bin Id	Marker Id	Gene Ids
Cokenii.contigs.canu	PF01653.13	tig00000152_7,tig00000295_19
Cokenii.contigs.canu	PF13241.1	tig00000292_370,tig00000292_462
Cokenii.contigs.canu	PF02617.12	tig00000113_6,tig00000292_400
Cokenii.contigs.canu	PF01351.13	tig00000021_18,tig00000296_539
Cokenii.contigs.canu	PF03710.10	tig00000297_83&&tig00000297_84,tig00000301_19
Cokenii.contigs.canu	PF02631.11	tig00000032_28,tig00000296_710
Cokenii.contigs.canu	PF00763.18	tig00000113_2,tig00000292_402
Cokenii.contigs.canu	PF11898.3	tig00000044_67,tig00000046_5
Cokenii.contigs.canu	TIGR01075	tig00000144_34,tig00000144_9,tig00000153_25,tig00000305_10
Cokenii.contigs.canu	PF01977.11	tig00000019_18,tig00000296_905
Cokenii.contigs.canu	PF07943.8	tig00000017_12,tig00000296_213
Cokenii.contigs.canu	PF09976.4	tig00000029_3,tig00000296_270
Cokenii.contigs.canu	PF01027.15	tig00000297_80,tig00000301_16
Cokenii.contigs.canu	TIGR01890	tig00000036_8,tig00000296_941
Cokenii.contigs.canu	PF01715.12	tig00000012_13,tig00000296_998&&tig00000296_999
Cokenii.contigs.canu	TIGR03594	tig00000029_1,tig00000296_268
Cokenii.contigs.canu	TIGR00244	tig00000022_17,tig00000296_433
Cokenii.contigs.canu	PF10385.4	tig00000113_19,tig00000292_397
Cokenii.contigs.canu	TIGR00639	tig00000022_1,tig00000296_454
Cokenii.contigs.canu	TIGR00633	tig00000032_1,tig00000296_693
Cokenii.contigs.canu	PF03379.8	tig00000019_31,tig00000296_917
Cokenii.contigs.canu	PF03119.11	tig00000152_5,tig00000295_17
Cokenii.contigs.canu	PF02938.9	tig00000142_13,tig00000297_139
Cokenii.contigs.canu	TIGR02521	tig00000029_6,tig00000296_273

**Bin Id**	**Marker Id**	**Gene Ids**
Cokenii.contigs.canu	PF00829.16	tig00000031_83,tig00000088_323
Cokenii.contigs.canu	PF07516.8	tig00000031_86,tig00000088_320
Cokenii.contigs.canu	PF02684.10	tig00000021_19,tig00000296_538
Cokenii.contigs.canu	PF05201.10	tig00000297_78,tig00000301_14
Cokenii.contigs.canu	PF01195.14	tig00000016_3,tig00000296_52
Cokenii.contigs.canu	TIGR01850	tig00000016_10,tig00000296_43
Cokenii.contigs.canu	TIGR00019	tig00000297_79,tig00000301_15
Cokenii.contigs.canu	TIGR02392	tig00000142_7,tig00000297_134
Cokenii.contigs.canu	PF00745.15	tig00000297_78,tig00000301_14
Cokenii.contigs.canu	PF01985.16	tig00000297_66,tig00000300_16
Cokenii.contigs.canu	PF04354.8	tig00000152_8&&tig00000152_9,tig00000295_21
Cokenii.contigs.canu	TIGR00214	tig00000017_10,tig00000296_215
Cokenii.contigs.canu	TIGR01189	tig00000019_30,tig00000296_916
Cokenii.contigs.canu	PF02660.10	tig00000044_175,tig00000297_230
Cokenii.contigs.canu	PF07717.11	tig00000044_67,tig00000046_5
Cokenii.contigs.canu	TIGR02012	tig00000032_30,tig00000296_711
Cokenii.contigs.canu	PF01411.14	tig00000032_26&&tig00000032_27,tig00000296_709

Supplementary Table S4 | Statistic of mapped raw PacBio reads on the NCBI nt database and *Chromatium okenii* str. LaCa genome and other PSB sequences from lake Cadagno. The Centrifuge *k*-mer classifier [12] was used to map all PacBio raw reads on the NCBI nt database that included also the *C. okenii* contigs and the draft genome of *Lamprocystis* CadA31 and the complete genome of "*Thiodictyon syntrophicum*" Cad16^T. A total of 96 % of all PacBio reads classified within the *Chromatiales* thereby mapped on the *C. okenii* contigs.

	# Reads	Percentage %
Raw reads	121,812	100
Classified reads	109,387	89.8
CladeReads classified to the Order *Chromatiales*	87,690	80.2
CladeReads classified on species level within the order *Chromatiales*		
Chromatium okenii	84,383	96.229
Lamprocystis purpurea	3,211	3.662
Thiocystis violascens	29	0.033
Thiodictyon syntrophicum	4	0.005
Thiocystis gelatinosa	1	0.001
others	29	0.033

Supplementary Table S5 | Phage and prophage sequences detected in the *Chromatium okenii* str. LaCa genome. *Phispy* [13] was used to detect prophage sequences. *VirSorter* [14] and *PHASTER* [15] were used to detect phage sequences in the *C. okenii* genome. Detected phages Chok1–5 were classified as putative by *VirSorter* and PHASTER classification of Chok6 was below a score of 70 (incomplete).

	Phage No.	Contig (accession)	Start	Stop	Length (bp)	No. of genes	Coverage (canu assembly)
detected prophages	pChok1	PPGH01000021.1	5,655	16,320	10,666	7	7.7
	pChok2	PPGH01000023.1	266	10,497	10,232	7	2.3
	pChok3	PPGH01000037.1	919,636	952,286	32,651	34	-
	pChok4	PPGH01000038.1	70,225	136,412	66,188	66	-
detected phages	Chok1	PPGH01000025.1	1	13,879	13,880	15	7.2
	Chok2	PPGH01000028.1	1	14,664	14,665	18	3.9
	Chok3	PPGH01000033.1	1	15,163	15,164	20	4.6
	Chok4	PPGH01000045.1	1	16,950	16,951	19	55
	Chok5	PPGH01000027.1	1	38,611	38,612	54	6.4
	Chok6	PPGH01000027.1	29,619	36,873	7,255	22	-

Supplementary Table S6 | Clustered Regularly Interspaced Short Palindromic Repeats (CRISPRs) found *Chromatium okenii* str. *LaCa*. *CRISPR-CAS++* [16] was used to identify CRISPR-Cas array in the *C. okenii* genome. Only CRISPR array with an evidence score above 1 were included in the table

Localization	Name	CRISPR start	CRISPR end	CRISPR length (bp)	DR consensus	DR length (bp)	No. of spacers
PPGH01000029.1	CRR1	7,720	8,161	442	GTCTTAATCCCCTTAAAAACGGGTCTCGATGCGAAC	36	6

Supplementary Table S7 | List of KEGG[17] -Classified enzymatic pathways for different Purple Sulphur Bacteria. *BLASTKoala* [17] was used to classify CDS according to the KEGG, EC: Enzyme Commission Number

KEGG map	Distinct ECs	*Chromatium okenii* str. LaCa	*Thiodictyon syntrophicum* str. Cad16^T	*Lamprocystis purpurea* str. CadA31	*Thiocystis violascens* str. DSM 198	*Allochromatium vinosum* str. DSM 180
Carbon fixation in photosynthetic organisms	25	12 (48.0 %)	18 (72.0 %)	18 (72.0 %)	17 (68.0 %)	17 (68.0 %)
Glycolysis / Gluconeogenesis	41	15 (36.6 %)	22 (53.7 %)	22 (53.7 %)	17 (41.5 %)	16 (39.0 %)
Glyoxylate and dicarboxylate metabolism	58	10 (17.2 %)	13 (22.4 %)	14 (24.1 %)	13 (22.4 %)	11 (19.0 %)
Phenylalanine, tyrosine and tryptophan biosynthesis	31	14 (45.2 %)	20 (64.5 %)	20 (64.5 %)	20 (64.5 %)	21 (67.7 %)
Reductive carboxylate cycle (CO_2 fixation)	13	7 (53.8 %)	9 (69.2 %)	9 (69.2 %)	8 (61.5 %)	7 (53.8 %)
Alanine, aspartate and glutamate metabolism	43	8 (18.6 %)	15 (34.9 %)	18 (41.9 %)	15 (34.9 %)	14 (32.6 %)
Arginine and proline metabolism	97	14 (14.4 %)	26 (26.8 %)	25 (25.8 %)	24 (24.7 %)	20 (20.6 %)
Citrate cycle (TCA cycle)	22	13 (59.1 %)	16 (72.7 %)	15 (68.2 %)	14 (63.6 %)	13 (59.1 %)
D-glutamine and D-glutamate metabolism	13	2 (15.4 %)	4 (30.8 %)	4 (30.8 %)	4 (30.8 %)	3 (23.1 %)
Fatty acid biosynthesis	21	7 (33.3 %)	10 (47.6 %)	10 (47.6 %)	10 (47.6 %)	9 (42.9 %)
Fatty acid metabolism	29	5 (17.2 %)	6 (20.7 %)	5 (17.2 %)	2 (6.9 %)	3 (10.3 %)
Tyrosine metabolism	63	7 (11.1 %)	8 (12.7 %)	9 (14.3 %)	6 (9.5 %)	7 (11.1 %)
Oxidative phosphorylation	12	7 (58.3 %)	9 (75.0 %)	9 (75.0 %)	9 (75.0 %)	9 (75.0 %)
Cysteine and methionine metabolism	64	15 (23.4 %)	20 (31.2 %)	22 (34.4 %)	18 (28.1 %)	16 (25.0 %)
Glycine, serine and threonine metabolism	57	12 (21.1 %)	16 (28.1 %)	17 (29.8 %)	16 (28.1 %)	18 (31.6 %)
Methane metabolism	33	3 (9.1 %)	11 (33.3 %)	10 (30.3 %)	6 (18.2 %)	7 (21.2 %)
Sulfur metabolism	30	7 (23.3 %)	10 (33.3 %)	10 (33.3 %)	9 (30.0 %)	8 (26.7 %)

Supplementary Figures

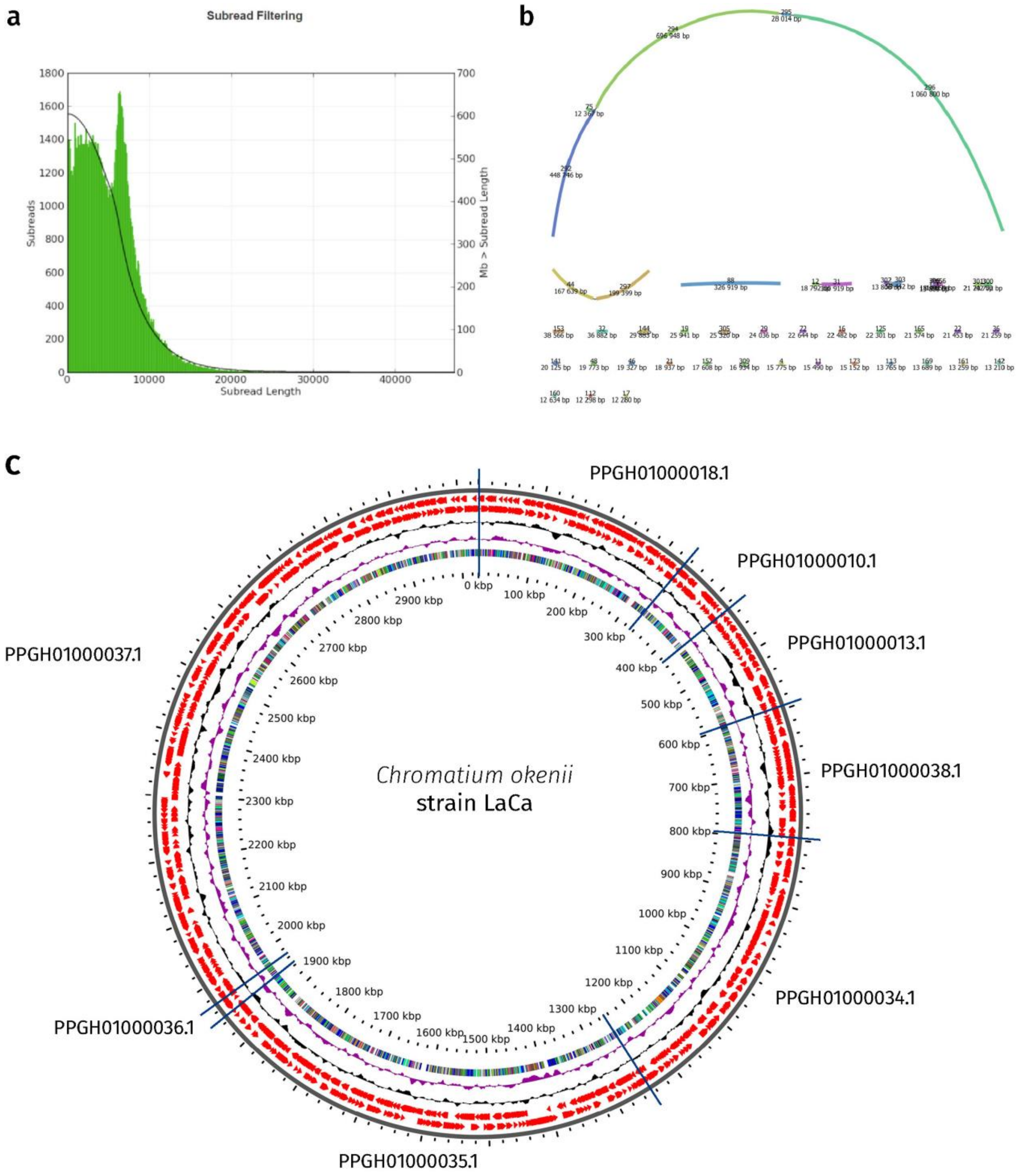

Supplementary Figure S1 | Graphical summary of the *Chromatium okenii* str. LaCa genome sequencing and assembly. a) Graph of the PacBio RSII output with the distribution of the read lengths after quality filtering. Sequencing of two SMRT cells resulted in total 611,754,382 bp, 95,063 reads with a N_{50} of 8,942 and a mean length of 6,435 bp after filtering. b) *canu* assembly scheme of the contigs of the *C. okenii* enrichment using the *bandage* package[18]. *BLASTn* [19]was used to align contiguous contigs and further assess possible overlaps at the contig ends. The 3.78 Mb chromosome encompasses eight contiguous contigs that could not be perfectly circularized due to long repetitive sequences. c) Pseudo-circular representation of the eight contigs that represent 90 % of the genome of *Chromatium okenii* str. LaCa. Red arrows; CDS on the positive and negative strand, black; GC content in %, violet: GC skew in %, CDS according to COG categories. The colour coding of the CDS represent different Clusters of Orthologous Groups categories. Borders between contigs are indicated with a blue stroke. *Gview* [20] was used to create Figure S1 c).

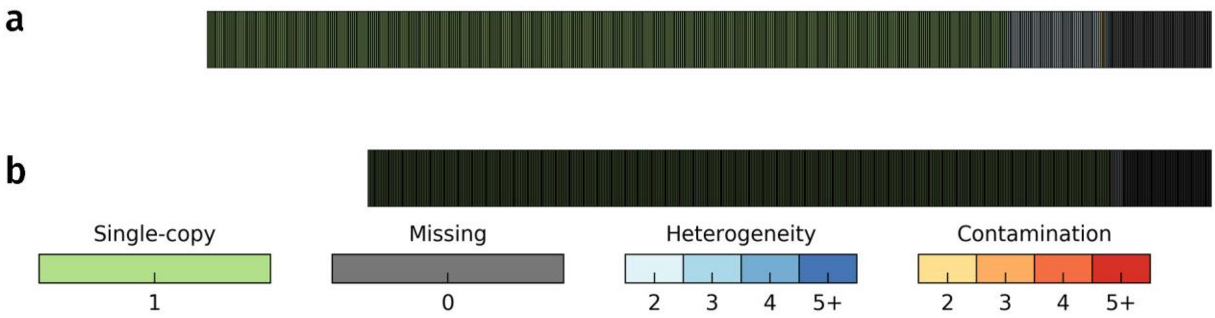

Supplementary Figure S2 | Graphical summary of genome completeness and contamination estimations for the *Chromatium okenii* str. LaCa genomic contigs. *CheckM* v1.0.12 [10] was used to asses genome quality. Plots were created with the `bin_qa_plot` command with default settings. a) When all 45 contigs are included 11 % contaminating sequences were found and strain heterogeneity was 95.24 %. b) Contaminating sequences are reduced to 1.1 % when only including the eight contiguous contigs and strain heterogeneity was reduced to 87.5 %. Completeness, contamination, and strain heterogeneity within each genome bin is depicted. As explained in the *CheckM* manual[11], green bars indicate unique markers, while bars in grey represent missing markers. Markers identified multiple times in a genome bin are represented by shades of blue or red depending on the amino acid identity (AAI) between pairs of multi-copy genes and the total number of copies present (2–5+). Pairs of multi-copy genes with an AAI ≥90 % are indicated with shades of blue, while genes with less amino acid similarity are shown in red. A gene present 3 or more times may have pairs with an AAI ≥90 % and pairs with an AAI < 90%.

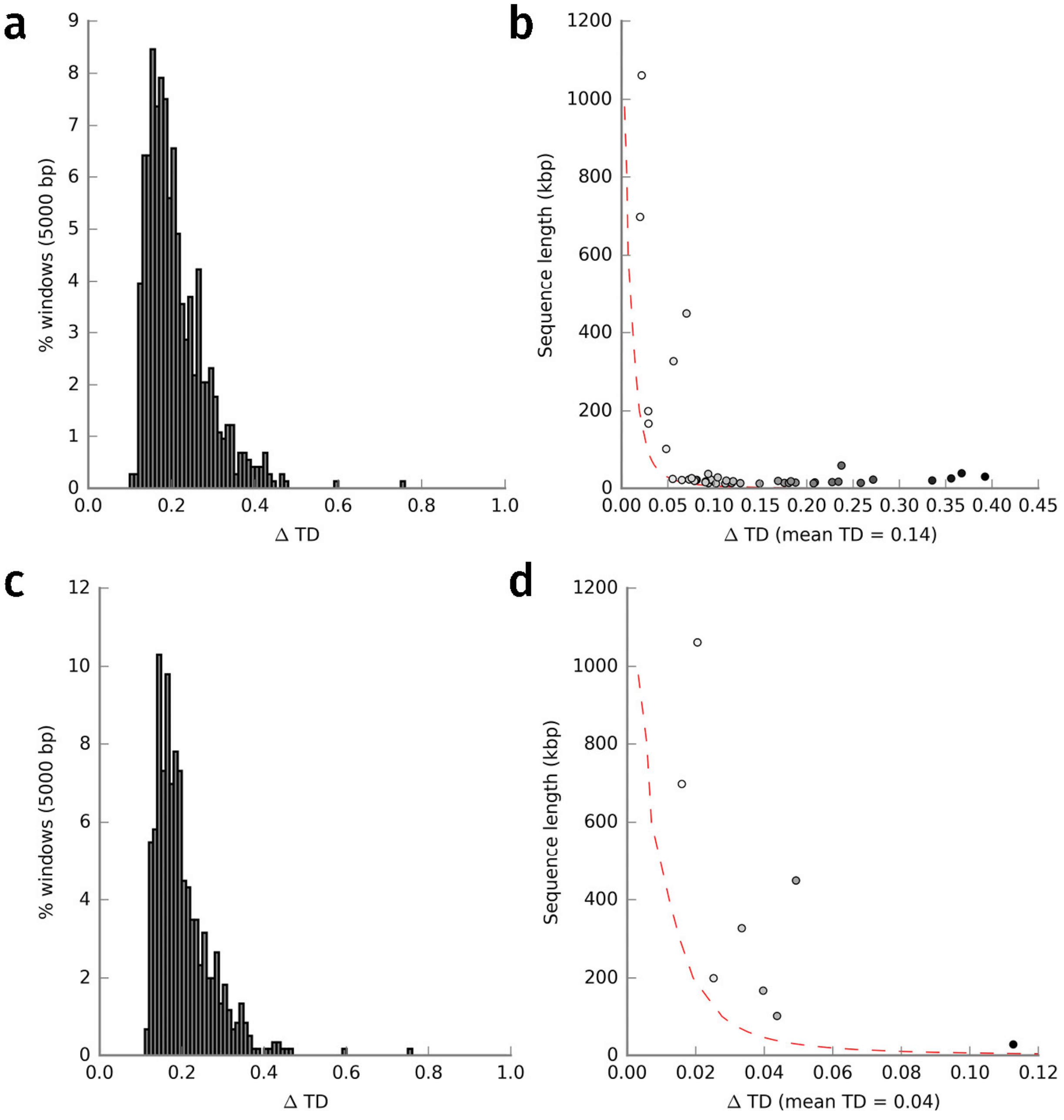

Supplementary Figure S3 | Graphical summary of the GC content (GC) and tetra-nucleotide frequency (TD) for the *Chromatium okenii* str. LaCa genomic contigs. *CheckM* v1.0.12 [10] was used to asses bin. Plots were created with the `dist_plot` command with default settings. a) The 45 contigs show unimodal distribution of the GC vs ΔTD values indicating no contaminating sequences. b) The seven longest contigs (PPGH01000037.1, PPGH01000035.1, PPGH01000034.1, PPGH01000018.1, PPGH01000038.1, PPGH01000013.1 and PPGH01000010.1) show a relatively lower ΔTD when compared to the residual contigs. The dashed red lines indicate the expected deviation from the mean GC as a function of length. This expected deviation is pre-calculated from a set of trusted reference genomes and the percentile plotted is provided as an argument to this command. c) When only the seven longest and PPGH01000036.1 are considered exclusively unimodal distribution is retained. d) PPGH01000036.1 markedly deviates from the seven longest contigs in ΔTD is however included in the chromosome linking the contig PPGH01000035.1 with PPGH01000037.1 as seen in supplementary Figure S1 b).

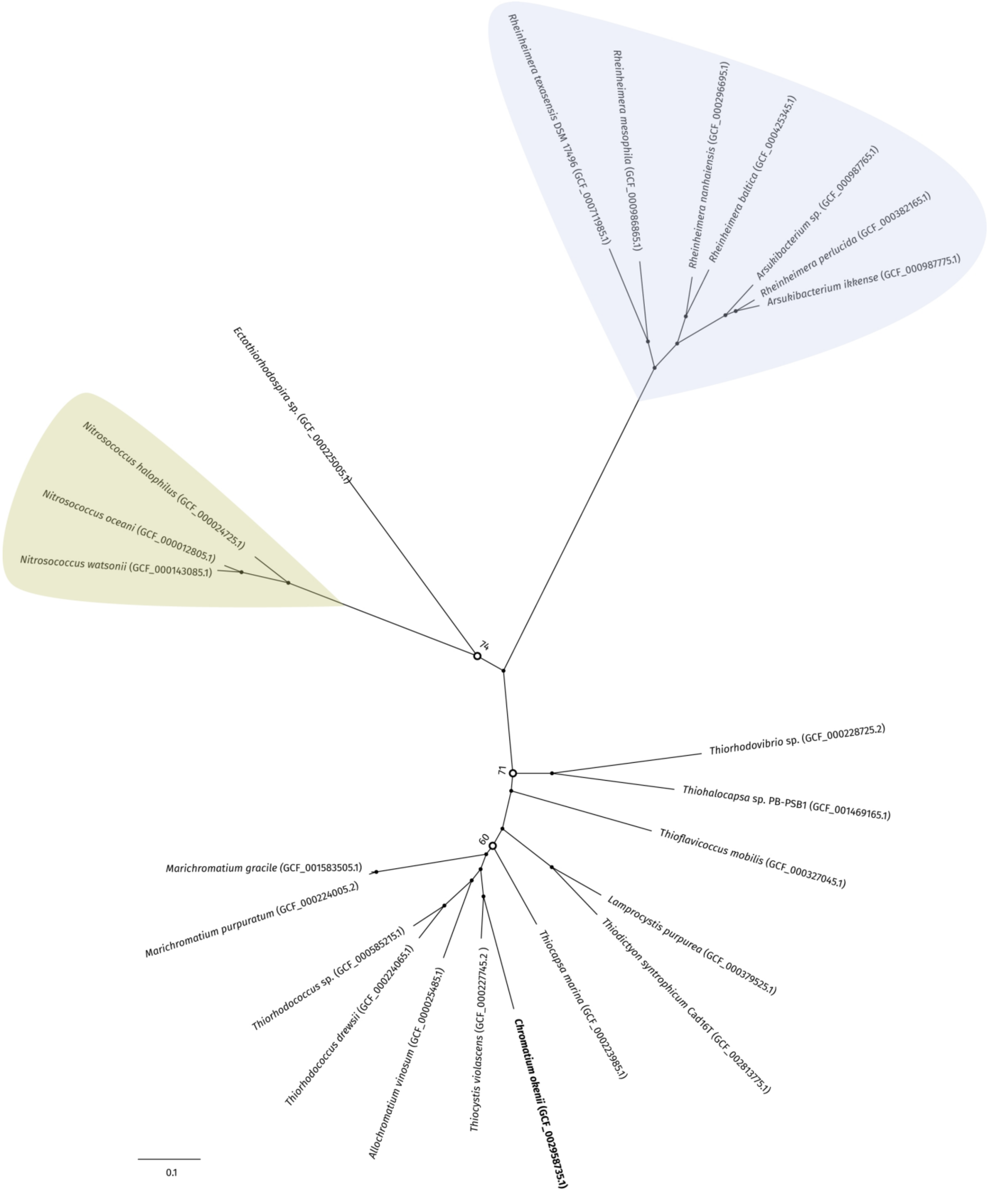

Supplementary Figure S4 | Phylogenetic tree including *Chromatium okenii* str. LaCa and 12 Chromatiaceae with WGS data available. Furthermore, the related phylogenetic lineages Nitrosococcus, Rheinheimera and Arsukibacterium are represented. Strain LaCa is most closely related to *Thiocystis violascens* DSM 198^T. *ROARY* [21] was used to identify single copy orthologues and *MUSCLE* [22] was used to align 100 randomly chosen sequences. *W-IQ-tree* [23] was used to infer phylogeny using the default settings. The consensus tree is based on best-model maximum-likelihood estimation and 1,000 bootstrap iterations. The node numbers indicate bootstrap support below 75%

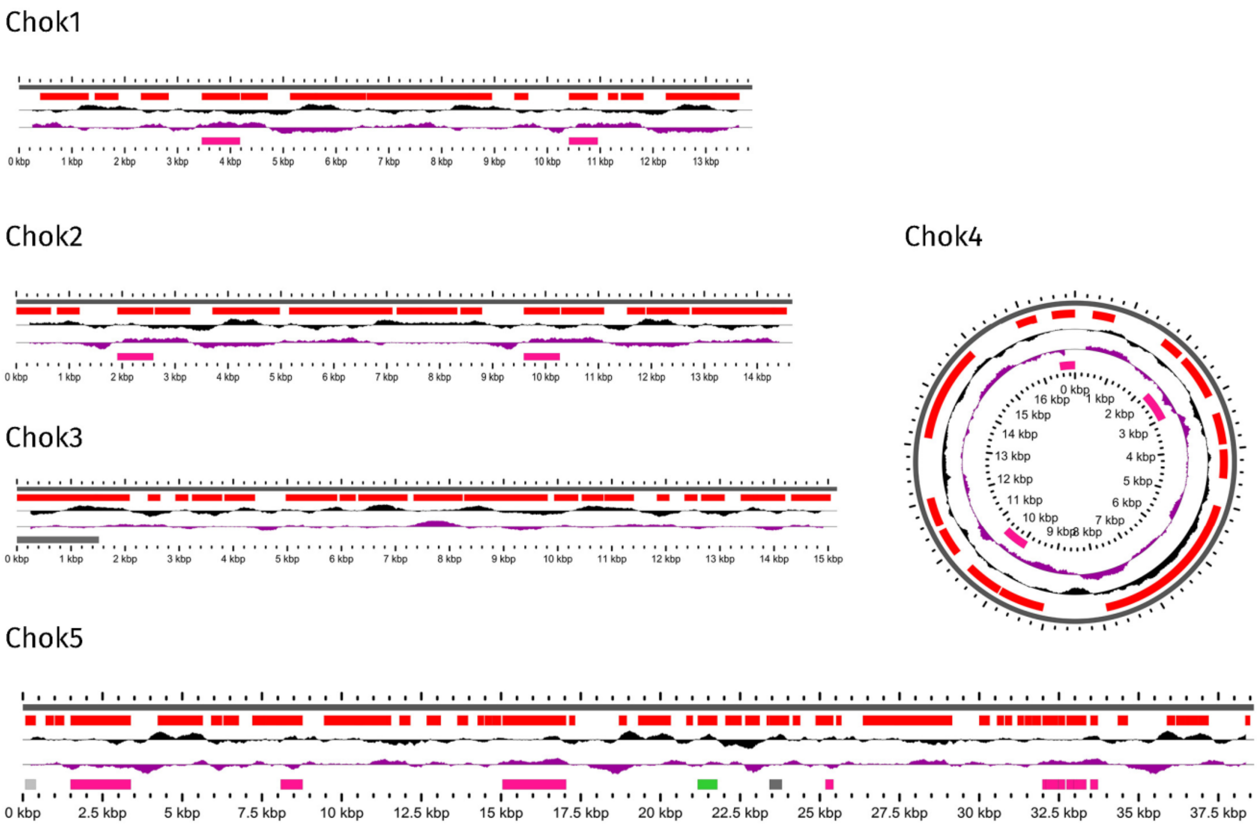

Supplementary Figure S5 | Genome maps of putative phage sequences found in the *Chromatium okenii* str. LaCa enrichment assembly. *VirSorter* [14]was used in the default mode to identify phage sequences**.** The sequences are rated as potential phages and comprise mainly hypothetical and phage-like proteins e.g. resolvases. Red blocks; CDS on both strands, black; GC content in %, violet: GC skew in %, CDS classified to COGs. The colour coding of the CDS represent different Clusters of Orthologous Groups categories. *Gview* was used to create Figure S5 [20].

References

1. Field, D. *et al.* The minimum information about a genome sequence (MIGS) specification. *Nat. Biotechnol.* **26**, 541–547 (2008).
2. Skerman, V. B. D., McGowan, V. & Sneath, P. H. A. Approved Lists of Bacterial Names. *Int. J. Syst. Evol. Microbiol.* **30**, 225–420 (1980).
3. Woese, C. R., Kandler, O. & Wheelis, M. L. Towards a natural system of organisms: proposal for the domains Archaea, Bacteria, and Eucarya. *Proc. Natl. Acad. Sci. U. S. A.* **87**, 4576–4579 (1990).
4. Perty, M. *Zur Kenntniss kleinster Lebensformen: nach Bau, Funktionen, Systematik, mit Specialverzeichniss der in der Schweiz beobachteten*. (Jent & Reinert, 1852).
5. Pfennig, N. *Chromatium okenii (thiorhodaceae): bio-convection aero and photoactic behaviour.* (Institut für den wissenschaftlichen Film, 1965).
6. Imhoff, J. F. The Family *Chromatiaceae*. in *The Prokaryotes* (eds. Rosenberg, E., DeLong, E. F., Lory, S., Stackebrandt, E. & Thompson, F.) 151–178 (Springer Berlin Heidelberg, 2014). doi:10.1007/978-3-642-38922-1_295
7. Trüper, H. G. CO2-Fixierung und Intermediärstoffwechsel bei *Chromatium okenii* Perty. *Arch. Für Mikrobiol.* **49**, 23–50 (1964).
8. Tonolla, M., Demarta, A., Peduzzi, R. & Hahn, D. *In situ* analysis of phototrophic sulfur bacteria in the chemocline of meromictic Lake Cadagno (Switzerland). *Appl. Environ. Microbiol.* **65**, 1325–1330 (1999).
9. Ashburner, M. *et al.* Gene Ontology: tool for the unification of biology. *Nat. Genet.* **25**, 25–29 (2000).
10. Parks, D. H., Imelfort, M., Skennerton, C. T., Hugenholtz, P. & Tyson, G. W. CheckM: assessing the quality of microbial genomes recovered from isolates, single cells, and metagenomes. *Genome Res.* **25**, 1043–1055 (2015).

11. CheckM - assessing the quality of genome bins. Available at: https://ecogenomics.github.io/CheckM/. (Accessed: 30th March 2016)

12. Kim, D., Song, L., Breitwieser, F. P. & Salzberg, S. L. Centrifuge: rapid and sensitive classification of metagenomic sequences. *Genome Res.* **26**, 1721–1729 (2016).

13. Akhter, S., Aziz, R. K. & Edwards, R. A. PhiSpy: a novel algorithm for finding prophages in bacterial genomes that combines similarity- and composition-based strategies. *Nucleic Acids Res.* **40**, e126–e126 (2012).

14. Roux, S., Enault, F., Hurwitz, B. L. & Sullivan, M. B. VirSorter: mining viral signal from microbial genomic data. *PeerJ* **3**, e985 (2015).

15. Arndt, D. *et al.* PHASTER: a better, faster version of the PHAST phage search tool. *Nucleic Acids Res.* **44**, W16–W21 (2016).

16. Couvin, D. *et al.* CRISPRCasFinder, an update of CRISRFinder, includes a portable version, enhanced performance and integrates search for Cas proteins. *Nucleic Acids Res.* **46**, W246–W251 (2018).

17. Kanehisa, M., Sato, Y. & Morishima, K. BlastKOALA and GhostKOALA: KEGG Tools for Functional Characterization of Genome and Metagenome Sequences. *J. Mol. Biol.* **428**, 726–731 (2016).

18. Wick, R. R., Schultz, M. B., Zobel, J. & Holt, K. E. Bandage: interactive visualisation of *de novo* genome assemblies. *bioRxiv* 018333 (2015). doi:10.1101/018333

19. Camacho, C. *et al.* BLAST+: architecture and applications. *BMC Bioinformatics* **10**, 421 (2009).

20. GView Server. Available at: https://server.gview.ca/examples. (Accessed: 12th October 2018)

21. Page, A. J. *et al.* Roary: rapid large-scale prokaryote pan genome analysis. *Bioinformatics* **31**, 3691–3693 (2015).

22. Edgar, R. C. MUSCLE: multiple sequence alignment with high accuracy and high throughput. *Nucleic Acids Res.* **32**, 1792–1797 (2004).

23. Trifinopoulos, J., Nguyen, L.-T., von Haeseler, A. & Minh, B. Q. W-IQ-TREE: a fast online phylogenetic tool for maximum likelihood analysis. *Nucleic Acids Res.* **44**, W232–W235 (2016).
